# Flavonoids as inhibitors of human neutrophil elastase

**DOI:** 10.1080/14756366.2021.1927006

**Published:** 2021-05-13

**Authors:** Katarzyna Jakimiuk, Jakub Gesek, Atanas G. Atanasov, Michał Tomczyk

**Affiliations:** a Department of Pharmacognosy, Faculty of Pharmacy with the Division of Laboratory Medicine, Medical University of Białystok, Białystok, Poland; b Department of Pharmacognosy, Medical University of Białystok, Student’s Scientific Association, Białystok, Poland; c Ludwig Boltzmann Institute for Digital Health and Patient Safety, Medical University of Vienna, Vienna, Austria; d Institute of Genetics and Animal Biotechnology of the Polish Academy of Sciences, Jastrzębiec, Poland; e Department of Pharmacognosy, University of Vienna, Vienna, Austria

**Keywords:** Flavonoids, elastase, inhibition, structure–activity relationship

## Abstract

Elastase is a proteolytic enzyme belonging to the family of hydrolases produced by human neutrophils, monocytes, macrophages, and endothelial cells. Human neutrophil elastase is known to play multiple roles in the human body, but an increase in its activity may cause a variety of diseases. Elastase inhibitors may prevent the development of psoriasis, chronic kidney disease, respiratory disorders (including COVID-19), immune disorders, and even cancers. Among polyphenolic compounds, some flavonoids and their derivatives, which are mostly found in herbal plants, have been revealed to influence elastase release and its action on human cells. This review focuses on elastase inhibitors that have been discovered from natural sources and are biochemically characterised as flavonoids. The inhibitory activity on elastase is a characteristic of flavonoid aglycones and their glycoside and methylated, acetylated and hydroxylated derivatives. The presented analysis of structure–activity relationship (SAR) enables the determination of the chemical groups responsible for evoking an inhibitory effect on elastase. Further study especially of the *in vivo* efficacy and safety of the described natural compounds is of interest in order to gain better understanding of their health-promoting potential.

## Introduction

The inhibition of human enzyme activity is an interesting strategy for treating global diseases and may be an attractive target for pursuing new drug discoveries^1^. Regulation of enzyme activity by elastase inhibitors is a promising endeavour for treating rheumatoid arthritis, glomerulonephritis, emphysema, pulmonary diseases, psoriasis, and cancers^2^^,^^3^.

Neutrophils are critical for the innate immune response; thus, they are involved in fighting infections. Neutrophil activation and degranulation lead to the release of serine proteases (elastase, proteinase 3, cathepsin G) into the extracellular space as proteolytically active enzymes that are capable of degrading a broad spectrum of extracellular matrix (ECM) proteins, such as fibronectin, elastin, or collagen, which provide physical support and stability to tissues^4–7^. Neutrophil-derived proteases, including elastase, have the ability to control the action of inflammatory cytokines by developing the immune response. However, human neutrophil elastase (HNE) is also able to intensify the emergence of other diseases^8–10^. HNE belongs to the chymotrypsin superfamily of serine proteases and is involved in the nonoxidative pathway of intracellular and extracellular pathogen destruction. Elastase is produced by human neutrophils, monocytes, macrophages, and endothelial cells and stored mainly in azurophilic granules and the nuclear envelope^11^^,^^12^. Under physiological conditions, HNE is counteracted by natural serine protease inhibitors, including elafin, *α*1-antitrypsin, and secretory leukocyte protease inhibitor (SLIP)^5^. Nevertheless, the protective role of endogenous inhibitors can be inactivated by the adhesion of neutrophils to the ECM, oxidants, and proteases produced by other leukocytes and by strongly linking HNE to receptors on the cell membrane, thus inhibiting the binding to accessible endogenous inhibitors^13^. Overall, the fluctuation in the quantity of HNE and its inhibitors plays a critical role in inducing a number of human diseases.

Although synthetic inhibitors are available, the identification of naturally derived drugs is a valuable research field for identifying inhibitors with a lack of unpleasant side effects. Among polyphenolic compounds, some flavonoids and their derivatives, which are mostly found in herbal plants, are potential inhibitors of elastase with few side effects. This review focuses on the diverse effects and efficacy of flavonoids and their derivatives in the development of elastase inhibitors.

## Methodology/search strategy

The search strategy helps to clarify the adequate search string and find the relevant subject databases to accurately identify appropriate scientific research. The search databases for this review were Taylor & Francis Online, Google Scholar, EBSCO Discovery Service (EDS), REAXYS Database, SCOPUS, PubMed/MEDLINE, Web of Science (SCI-EXPANDED), Wiley Online Library, and Science Direct/ELSEVIER. For the review method, the above databases were searched using different combinations of the following keywords: elastase, neutrophil, biological functions, elastase activity, serine protease, infection, inhibitor, flavonoids, human disorders, enzyme, biological activity, and immune response.

### Biological functions of human neutrophil elastase

In adult mammalian organisms, neutrophils are produced in the bone marrow and released into blood and tissues under certain physiological conditions. The human body makes over 1 billion neutrophils per day/kg body weight. Nevertheless, during various autoimmune and inflammatory diseases, their number can expand to 10 billion. In an inflammatory environment, neutrophils can survive for seven days, which may be connected with cytokine-activated endothelial cell action^14^. They are the first line of defence against bacterial and fungal infections and help combat parasites and viruses. Their diverse functions include protection against reactive oxygen species (ROS) and hydrolytic enzymes and elimination of pathogens, thus making them an important part of the overall immune and inflammatory response (phagocytosis, degranulation, and NETosis). On the other hand, this type of leucocyte is capable of contributing to tissue damage during various autoimmune and inflammatory diseases and plays important roles in various pathologies^15^.

One of the neutrophil functions is to produce and release serine proteases (elastase, proteinase 3, and cathepsin G). HNE is known to play multiple roles in the human body. Elastase is a cytotoxic 29-kDa protease, and sequence analysis has demonstrated that it consists of polypeptides with single chains and 218 amino acids with four intramolecular disulphide bonds linking eight half-cystine residues^16^^,^^17^.

Enzymes are released to defend against invading pathogens via their ability to control apoptosis^18^^,^^19^. The mechanism of action of neutrophil elastase (NE) is based on cleaving bacterial virulence factors and their outer membrane proteins and binding to the bacterial membrane^10^. Furthermore, HNE is involved in the inflammatory response by inducing interleukin 8 (IL-8) through Toll-like receptor 4 (TLR4) activation and the release of other proinflammatory cytokines^20–22^. This enzyme may also cause degradation of elastic fibres and induce proliferation of keratinocytes^23^^,^^24^. Other biological functions of HNE are given in Table 1.

**Table 1.** Biological functions of HNE.

Elastase functions	Model of the study	References
Bactericidal ability	The respiratory tract cells	^18^
Control of apoptosis and participation in phagocytosis		
Role in mucin production		
Bioactivity and ability to control some inflammatory cytokines	Membrane-bound human leukocyte elastase	^4^^,^^19^^,^^25^
Cleaves immunoglobulins, complement components, complement receptor type 1 on neutrophils	Human neutrophils	^26^^,^^27^
Participates in cell differentiation, migration, and angiogenesis	Extracellular matrix	^28^
Induces IL-8 expression by activating TLR4 and degrading components of the lung matrix	Bronchial epithelium	^20^
Cleaves receptors and lung surfactant protein	Animal models	^11^
Increases PAR2 expression and mucin5ac protein release in mucus hypersecretions	Epithelial cells	^29^
Regulates lung endothelial cell barrier integrity through proteinase-activated receptor (PAR1)	Endothelial cells	^30^^,^^31^
Stimulates airway submucosal gland secretion	*In vitro* and *in vivo* mice model	^32^
Promotes the neutrophil-mediated activation of platelets	Platelets	^33^
Induces proliferation of keratinocytes in tissue repair	*In vitro* on murine keratinocyte cell line; *in vivo* on mice skin	^24^
Degenerates elastic fibres in tissue repair	*In vivo* on mice skin	^23^

### Role of HNE in infections

The main goal of the innate immune response is to locate and destroy pathogens that have entered the human body. One of the first immune system components that reaches the site of infection is neutrophils. They fight pathogens through non-specific immune mechanisms with the help of ROS and enzymes that are involved in oxidative and nonoxidative defence pathways. HNE is one of the critical factors in the innate immune system with antimicrobial activity. Neutrophil elastase can be activated by cathepsin-C, and then the enzyme is involved in many nonoxidative immune responses^34^.

Phagocytosis is a defence mechanism against pathogens. It is an intracellular process initiated by the binding and recognition of pathogens through cell membrane receptors that are subsequently absorbed into structures called phagosomes^35^. Afterwards, the granules are attached to the absorbed phagosome and shed its contents, and the resulting phagolysosome starts the degradation of the absorbed pathogen. NE kills microbes, e.g. *Escherichia coli*, by degrading the outer membrane protein A (OmpA), which disrupts the cell membrane integrity and leads to subsequent death^36^. Moreover, the simultaneous action of serine proteases led to the death of gram-positive *Streptococcus pneumoniae* during phagocytosis *in vivo*^37^.

Another way of fighting microbes that requires elastase is via degranulation. Unlike phagocytosis, this process shows activity in the ECM. The stimulation of neutrophils by cytokines leads to the transfer of granularity to the cell periphery, where the granules are fused with the cell membrane and their content is poured out of the cell^11^. The primary granule content is targeted at the pathogen killing process. However, they are released due to their high toxicity and simultaneously there is a high possibility of damage to surrounding tissues^38^^,^^39^. Extracellular HNE shows a cleavage effect on many bacterial proteins, e.g. leukotoxins, which is a factor leading to the lysis of leukocytes^40^^,^^41^.

**Figure 1.**
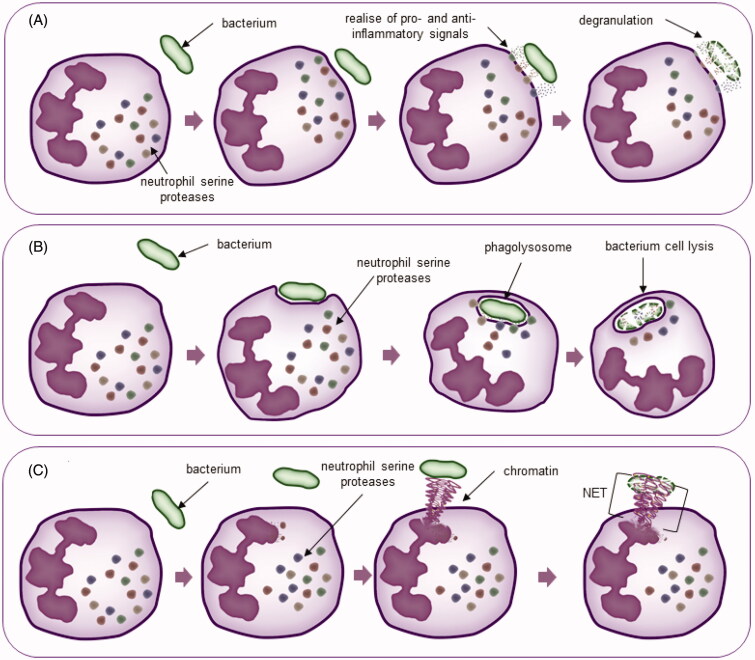
Neutrophil mechanisms of action. (A) Degranulation; (B) phagocytosis; (C) NETosis.

Moreover, HNE demonstrates its activity in NETosis, a mechanism used by neutrophils to tackle pathogens. NETosis is a complex of decondensed and unfolded DNA with histones and cytoplasmic granule proteins^42^. Induction by IL-8 and lipopolysaccharide (LPS) leads to the activation of neutrophils, which contain proteolytic enzymes. Thus, in this process, NETs are involved in fighting the infection because NE is one of the factors affecting the release of DNA from its condensed form. Elastase is transported to the nucleus, and its enzymatic activity is a determinant of the degradation of histones, which promotes the release of DNA^43^. Additionally, elastase presence in NETs is a destructive factor for yeasts, hyphal forms of fungi, e.g. *Candida albicans*, and bacteria, e.g. *Shigella flexneri*^42^^,^^44^ (Figure 1).

### Role of neutrophil elastase inhibitors in human diseases

Extended increases in the activity of HNE may cause tissue destruction that is linked with infections and inflammation. Thus, HNE functions are involved in a variety of severe chronic diseases, particularly respiratory, urinary, integumentary, digestive, reproductive, nervous, and skeletal pathologies (Table 2).

**Table 2.** HNE in human disorders

System	Type of disorder	Model used in the study	References
Respiratory	Acute lung injury (ALI)	*In vitro* and *in vivo* studies, both clinical and animals models	^22^^,^^45^
	Severe pneumonia	Clinical features in adult patients	^46^
	Acute respiratory distress syndrome (ARDS)	*In vitro* and *in vivo* studies	^4^
	Asthmatic exacerbations		
	Pulmonary fibrosis		
	Adult respiratory distress syndrome	Epithelial cells in the respiratory system	^47^
	Chronic bronchitis		
	Viral- or pollution-triggered asthma		
	Chronic obstructive pulmonary disease (COPD)	Clinical and pre-clinical trials	^26^^,^^48^
	Smoke-induced pulmonary emphysema	Mice	^49^
	Chronic obstructive airways disease (COAD)	Clinical trials	^50^
	Ventilator-induced lung disease	Mutant neonatal mice	^51^
	Metastasis formation of lung cancer	Immunodeficiency mice	^52^
	Bronchiolitis obliterans syndrome	*In vitro* and *in vivo* studies	^53^
Urinary	End-stage renal disease (ESRD)	*In vitro* and *in vivo* studies, clinical trials	^54^
	Chronic kidney disease		
	Glomerulonephritis		^50^
Integumentary	Chronic skin ulceration	Skin cells	^3^
	Bullous pemphigoid	Mice	^55^
	Papillon-Lefèvre syndrome	*In vitro* and *in vivo* studies, clinical trials	^11^
	Psoriasis	*In vitro* and *in vivo* studies	^53^
Digestive	Inflammatory bowel disease	Mice	^56^
Reproductive	Metastasis formation of human breast cancer	Immunodeficient mice	^53^
	Prostate cancer	*In vitro* and *in vivo* studies	
Skeletal	Rheumatoid arthritis	*In vitro* and *in vivo* studies	
Immunity	Graft-versus-host disease	Pre-clinical trials	^57^

For example, alvelestat (MPH-966), an oral NE inhibitor, has adverse effects on 5-FU-induced intestinal mucositis in patients with colorectal cancer by controlling aberrant inflammatory responses, intestinal barrier dysfunction, and gut microbiota imbalance^58^. It is worth mentioning that sivelestat (ONO-5046), another HNE inhibitor, might be useful as a potent drug for the treatment of acute lung injury, acute respiratory distress syndrome or coagulopathy in patients with COVID-19^17^^,^^59^. Moreover, this selective NE inhibitor could be considered for its role in suppressing excessive inflammation post-myocardial infarction and apoptosis and preventing left ventricular remodelling in a mouse model^60^. ONO-5046 also limited the incidence of collagen-induced arthritis in rat and mouse models^61^ and prevented bleomycin-induced pulmonary fibrosis in mice^62^. It has been reported that after the administration of other elastase inhibitors, such as ZD-0892 and M249314 (peptidyl trifluoromethyl ketones), pulmonary artery pressure and muscularisation were reduced when used in clinical trials^63^.

Furthermore, elastase is able to damage the integrity of the ECM barrier, which can directly cause cancer expansion^7^.

### Inhibitory effect of flavonoids on elastase activity

Phenolic compounds represent a large percentage of the secondary metabolites of diverse plants. Thus, flavonoid aglycones and glycosides remain one of the most extensive groups of polyphenols in the plant kingdom. Flavonoids consist of two benzene rings and one heterocyclic pyran ring, which can be divided into subgroups depending on the point of attachment of the B-carbon ring to the C-carbon ring and the degree of its oxidation and according to their chemical substitutions^64^^,^^65^. Due to the significant role of NE in the healing process and the development of rheumatoid arthritis, glomerulonephritis, emphysema, pulmonary diseases, psoriasis, and even cancers, several studies have reported the identification of elastase inhibitors from natural sources. Plants producing secondary metabolites and phytochemicals have great potential to act as therapeutics^2^^,^^3^^,^^66^. The elastase inhibitory activity of many plant extracts and compounds has been investigated to identify new sources of anti-elastase drugs. A wide range of flavonoid compounds, including aglycones and their *O*- and *C*-glycosides, were investigated for their potential elastase inhibitory activity (Table 3).

**Table 3.** Flavonoids measured for anti-elastase activity and their respective IC_50_ values.

Tested compound	IC_50_ value	References
Luteolin	>300 µM	^67^
	12 µM	^68^
	8.06 ± 2.73 μM	^69^
	12.7 ± 0.5 μM	^70^
	6.91 μM	^71^
	36.01 ± 1.15 μM	^72^
	7.65 ± 0.77 μM	^73^
Luteolin 4′-*O*-*β*-d-glucoside	13.72 ± 5.26 μM	
Luteolin 4′-methylether	4.13 ± 0.47 μM	^74^
Luteolin 7-*O*-*β*-d-glucoside	No significant inhibitory activity	^73^^,^^75^
Luteolin 8-C-glucoside	146.1 ± 38.8 μM	^76^
Apigenin	27.6 ± 1.0 µg/mL	
	46.1 ± 0.9 µM	^70^
	37.94 ± 2.06 µM	^72^
	13.35 ± 0.37 μM	^73^
Apigenin 4′-*O*-*β*-d-glucoside	No significant inhibitory activity	^77^
	>23.13 μM	^73^
Apigenin 7-*O*-*β*-d-glucoside	No significant inhibitory activity	
Apigenin 7-*O*-rhamnoglucoside	>10 µM	^78^
Apigenin 8-C-glucoside	120.95 ± 10.6 μM	^76^
Apigenin 6-C-glucoside	4.34 ± 0.58 µM	^79^
Baicalein	2.2 µM	^68^
	3.53 µM	^80^
	25 µM	^67^
	No significant inhibitory activity	^81^
Baicalein 6,7-di-*O*-methyl	>10 µM	^82^
Baicalein 7-*O*-methylether		
6-Hydroxy-5,7-dimethoxyflavon		
Diosmetin 7-*O*-rutinoside	>16.43 μM	^73^
Chrysin	2.44–0.09 µM	^82^
	6.7 µM	^68^
	No significant inhibitory activity	^61^
Norartocarpetin	>300 µM	^83^
Cupressuflavone	8.09 ± 0.92 µM	^84^
Amentoflavone	1.27 ± 0.16 µM	
	0.75 ± 0.18 µM	^85^
Robustaflavone	1.33 ± 0.21 µM	^84^
	0.45 ± 0.11 µM	^85^
Rhusflavanone	19.54 ± 2.4 μM	^76^
Mesuaferrone B	19.06 ± 2.4 μM	
Tricin	17.69 ± 1.71 µM	^86^
4′-*O*-Geranyltricin	12.80 ± 6.84 µM	
3′-*O*-Geranylpolloin	17.34 ± 3.81 µM	
Velutin	4.26 ± 0.12 µM	
Afrormosin	No significant inhibitory activity	^87^
Boeravinone T		^88^
Boeravinone B		
Boeravinone U		
Boeravinone J		
Boeravinone X		
Hypolaetin 7-*O*-*β*-xyloside	>100 µM	^84^
6,8-Diprenylorobol	1.3 ± 0.3 µM	^89^
5,7,3′,4′-Tetrahydroxy-2′,5′-di(3-methylbut-2-enyl)isoflavon	213.1 ± 1.9 µM	
Flemiphilippinin A	8.3 ± 0.4 µM	
5,7,3′-Trihydroxy-2′-(3-methylbut-2-enyl)-4′,5′-(3,3-dimethylpyrano)isoflavone	22.4 ± 0.7 µM	
8-γ,γ-Dimethylallylwighteone	6.0 ± 0.3 µM	
Osajin	26.0 ± 0.6 µM	
Flemingsin	12.0 ± 0.4 µM	
Flemichin D	5.3 ± 0.5 µM	
Lupinifolin	13.3 ± 0.1 µM	
Khonklonginol H	110.2 ± 0.8 µM	
Auriculasin	3.1 ± 0.2 µM	^11^
Orobol 7,3′-di-*O*-methyl ether	>10 µM	^85^
Genistein	25.87 ± 5.99 μM	^73^^,^^82^
	51.4 ± 0.5 µM	^89^
	63 µM	^90^
	42.15 ± 2.88 µM	^79^
Daidzein	4.29 ± 0.49 µM	
Vigvexin A	17.27 ± 4.19 µM	
Vigvexin B	12.62 ± 7.17 µM	
5,7,4′-Trihydroxy-3′-methoxy isoflavone	19.37 ± 4.16 µM	
Quercetin	5.51 ± 1.07 µM	
	14.3 ± 0.2 µM	^70^
	2.6 µM	^68^
	1.5 µM	^91^
	334.18 ± 3.3 μM	^92^
	20 µM	^67^
	2.65 μM	^92^^,^^93^
Quercetin 7-*O*-methylether	18.3 µM	^68^
Quercetin 3-*O*-rhamnoside	113.29 ± 1.9 μM	^76^
	36.98 ± 9.1 μM	^81^
Quercetin 3-methylether	19 µM	^94^
Quercetin 3,3′-dimethylethe	129 µM	
Quercetin 3-*O*-rutinoside	6.9 µM	^91^
	9.8 µM	^68^
Quercetin 3-*O*-galactoside	0.3 µM	
	0.32 μM	^93^
	1.94 μM	^95^
Quercitrin	11.1 µM	^68^
	>100 µM	^84^
Isoquercitrin		
	1.4 µM	^68^
	1.5 μM	^93^^,^^95^
Quercetagetin 3,6-dimethylether	115 µM	^94^
Fisetin	16 µM	^67^
Myricetin	4 µM	
	21.1 µM	^68^
Myricetin 3-*O*-rhamnoside	No significant inhibitory activity	^84^
Morin 3-*O*-*α*-rhamnoside	8.52 ± 0.18 μM	^96^
Morin	4.5 µM	^67^
	11.6 µM	^68^
Naringenin	84 µM	
Vitexicarpin	>10 µM	^78^
Ugonin M	1.6 ± 0.33 µM	^97^
Ugonin O	3.4 ± 0.50 µM	
Ugonin Q	0.49 ± 0.27 µM	
Ugonin R	4.56 ± 0.32 µM	
Ugonin S	1.9 ± 0.52 µM	
Ugonin T	1.2 ± 0.13 µM	
Ugonin K	>10 µM	
Ugonin L	3.8 ± 0.08 µM	
Kaempferol	5000 µM	^67^
Kaempferol 6-hydroxy-3,6-dimethylether	194 µM	^94^
Kaempferol 3,7-dimethylether	61 µM	
6,8-Diprenylkaempferol	29.3 ± 0.3 µM	^89^
Kaempferol 3-*O*-*α*-rhamnoside	>100 µM	^84^
	154.71 ± 6.48 μM	^76^
	38.09 ± 12.19 μM	^96^
Kaempferol 3-*O*-*α*-glucoside	19.20 ± 3.08 μM	
	142.28 ± 6.24 μM	^76^
Kaempferol 3-*O*-rutinoside	>100 µM	^91^
Formononetin 7-*O*-glucoside	>232 μM	^98^
Sativanone 7-*O*-glucoside	>215 μM	
Eriodictyol 7-*O*-rutinoside	>400 µM	^68^
2-(3,4-Dihydroxy-2-[(2,6,6-trimethylcyclohex-2-enyl)-methyl]phenyl)-3,5,7-trihydroxy-4H-chromen-4-one	0.98 ± 0.15 µM	^99^
2-(3,4-Dihydroxyphenyl)-6-((2,2-dimethyl-6-methylenecyclohexyl)-methyl)-5,7-dihydroxy-chroman-4-one	>10 µM	
4″a,5″,6″,7″,8″,8″a-Hexahydro-5,3′,4′-trihydroxy-5″,5″,8″a-trimethyl-4H-chromeno[2″,3″:7,8]flavone	2.50 ± 0.37 µM	
4″a,5″,6″,7″,8″,8″a-Hexahydro-5,3′,4′- trihydroxy-5″,5″,8″a-trimethyl-4H-chromeno[2″,3″:7,6]flavone	>10 µM	
7-Hydroxy-6-methoxy-2-(2-phenylethyl)chromone	3.91 ± 0.87 µM	^86^
5-Hydroxy-7,3′,4′-trimethoxyflavon	9.32 ± 1.37 µM	
6,7-Dimethoxy-2-(2-phenylethyl)chromone	10.48 ± 1.35 µM	
(2R, 3R)-6-methyl-3′-geranyl-2,3-trans-5,7,4′-trihydroxy-flavonol	17.9 ± 1.5 µM	^100^
(E)-3-(3-(3,7-dimethylocta-2,6-dienyl)-2,4-dihydroxyphenyl)-3,5,7-trihydroxy-chroman-4-one	8.4 ± 0.8 µM	
3′-Geranyl-5,7,2′,4′ tetrahydroxyisoflavanone	30.8 ± 1.3 µM	

It has been reported that the 3-*O*-*β*-d-glucuronides of myricetin, mearsetin, quercetin, isorhamnetin, kaempferide, and kaempferol, the 3-*O*-*β*-2″-*O*-acetyl-*β*-d-glucuronides of kaempferol, isorhamnetin, and the 3-*O*-*β*-3″-*O*-acetyl-*β*-d-glucuronides of quercetin and kaempferol significantly decrease the release of elastase by neutrophils at a concentration of 1 μM^101^. In another chemical and biological study, extracts from aerial parts of *Hedysarum coronarium* L. with a high concentration of quercetin and tannins revealed dose-dependent inhibitory properties^102^.

Breviscapine, a flavonoid obtained from *Erigeron breviscapus* reduces NE levels associated with pulmonary inflammatory response and lung function in children undergoing open-heart surgery. A positive effect was observed in patients taking 1 mg/kg or 0.5 mg/kg breviscapine^103^. Compounds isolated from the ethyl acetate extract of *Scorzonera latifolia* were also selected for further investigation of their inhibitory effect. Quercetin 3-*O*-*β*-apiofuranosyl-(1‴→2″)-*β*-d-glucoside and 7-methylisoorientin display anti-elastase activities of 30.16% and 28.60%, respectively^104^. Phloretin obtained from *Malus doumeri* var. *formosana* has been shown to inhibit elastase in a concentration-dependent manner. At concentrations of 36.5–366 µM, 51.8–77.3% enzyme inhibition was observed^105^^,^^106^. The flavonone sakuranetin at a concentration of 100 µM reduces the release of elastase by 60%^107^. In a different study, sakuranetin was applied in an *in vivo* mouse model and did not show adverse clinical effects in preventing elastase-induced emphysema^108^. A 7-*O*-methylaromadendrin isolated from *Inula viscosa* decreased elastase production by 50% at 100 µM^107^. Glycitin was also evaluated for its NE release inhibitory properties, and the results revealed that a compound at 10 µM lowered enzyme activity^109^. 5-*O*-demethylnobiletin, a polymethoxyflavone isolated from *Sideritis tragoriganum*, inhibited elastase release by 48% at 10 µM. It is worth mentioning that the described flavonoids did not affect the activity of this enzyme^110^. The results of the elastase assays showed that at a concentration of 100 µM, naringenin, liquiritigenin, quercetin, apigenin, and sulfuretin possess inhibitory activities of 39%, 52%, 65%, 57%, and 38%, respectively^111^. The elastase inhibitory activities of the isolated compounds from the EtOAc (ethyl acetate) subextract of *Epilobium angustifolium* were also evaluated. Hyperoside, kaempferol, kaempferol 3-*O*-*α*-l-rhamnoside, quercetin 3-*O*-*α*-l-rhamnoside, and quercetin 3-*O*-*α*-l-arabinoside at a concentration of 100 µg/mL revealed inhibitory potentials of 19.87%, 15.33%, 9.76%, 8.92%, and 7.08%, respectively^112^.

The inhibitory effect of water–ethanol extract obtained from *Cecropia pachystachya* leaves, which has a total flavonoid content of 72.71 µg QE/mg DE, began at 0.8 µg/mL (15.79% elastase inhibition) and notably increased at 4 µg/mL (41.44%), 8 µg/mL (55.45%), and 16 µg/mL (50.99%)^113^. The anti-elastase activity of aqueous extracts from the leaves of *Ligustrum vulgare* L. was determined based on the contents of the flavonoids aglycones and glycosides (luteolin glucoside, quercetin rutinoside, and ligustroflavone). The aqueous extract at concentrations ranging from 5 µg/mL to 50 µg/mL inhibited HNE release by 23.9–34.1%^114^. It is worth mentioning that an ethanol extract of *Aceriphyllum rossii* leaves, which has a total flavonoid content of 206.3 mg/g, exhibits 99.2% inhibition at 10 mg/mL *in vitro*^115^. Fermenting red ginseng (FRG) was investigated as a novel skin-care antiaging ingredient based on its elastase inhibition potency. FRGs consist of 133.2 µg/mL flavonoid compounds, which may be connected with the IC_50_ value (117.07 µg/mL) for elastase inhibitory activity^116^. The leaf hydroalcoholic extract (EDE) from *Eugenia dysenterica* was characterised to determine its quercetin and other phenolic contents, and EDE was capable of inhibiting elastase in a dose-dependent manner at 25–100 µg/mL, with 45% activity observed at a concentration of 100 µg/mL^117^.

Many authors have identified anti-elastase activity based on EC_50_ values. *Meum athamanticum*, *Centella asiatica*, and *Aegopodium podagraria* water–glycerin extracts are described by a high amount of flavonoid compounds and demonstrate EC_50_ (%) values of inhibitory activity at 0.92, 0.52, and 1.03, respectively^118^.

The extracts obtained by subcritical water extraction from the stems, leaves, and berries of *Aronia melanocarpa* also reveal anti-elastase potential. At this stage, researchers determined both the total phenolic and total flavonoid contents. The leaves had the highest total phenolic and flavonoid contents, followed by the stems and berries, with 131.53 mg CAE/g extract, 49.96 mg CAE/g extract, and 13.88 mg CAE/g extract for phenolics, respectively, and 88.64 mg RE/g extract, 25.10 mg RE/g extract, and 10.00 mg RE/g extract for flavonoids, respectively. Moreover, flavonoids constitute over 70% of all phenolic compounds in aronia berries. All *A. melanocarpa* extracts expressed elastase inhibitory activity, with the highest potential observed in berry extracts (3.549 ± 0.113 mmol CAE/g extract)^119^. According to the LC–MS analysis, *Libidibia ferrea* bark and pod extracts are the sources of rutin, quercetin, kaempferol, apigenin, isorhamnetin, and taxifolin, and the samples showed approximately 36% elastase inhibition at 250 µg/mL for bark extract and 20% for pod extract^120^. Three flavonoids were isolated from the ethyl acetate fraction of the *Alchornea cordifolia* leaves: quercetin, myricetin 3-glucoside, and myricetin 3-rhamnoside. The anti-elastase activity was evaluated for aqueous and ethyl acetate extracts in cell-free and cellular models. In an acellular system, the IC_50_ values reached 4.7 and 2.2 mg/L for aqueous and ethyl acetate extracts, respectively. In a cellular model, polymorphonuclear neutrophils were stimulated by PMA (4*β*-phorbol-12-myristate-13-acetate), CaI (calcium ionophore), and fMLP (N-formyl-methionyl-leucine-phenylalanine). The IC_50_ values in the stimulated cellular experiment were in the range of 5.9–8.6 mg/L in the ethyl acetate extract and 7.3–12.1 mg/L in the aqueous extract. Among the ethyl acetate and aqueous extracts, the more active extract was the ethyl acetate, which may be connected with its higher content of flavonoids^121^.

### Flavonoid structure–activity relationship (SAR)

**Figure 2.**
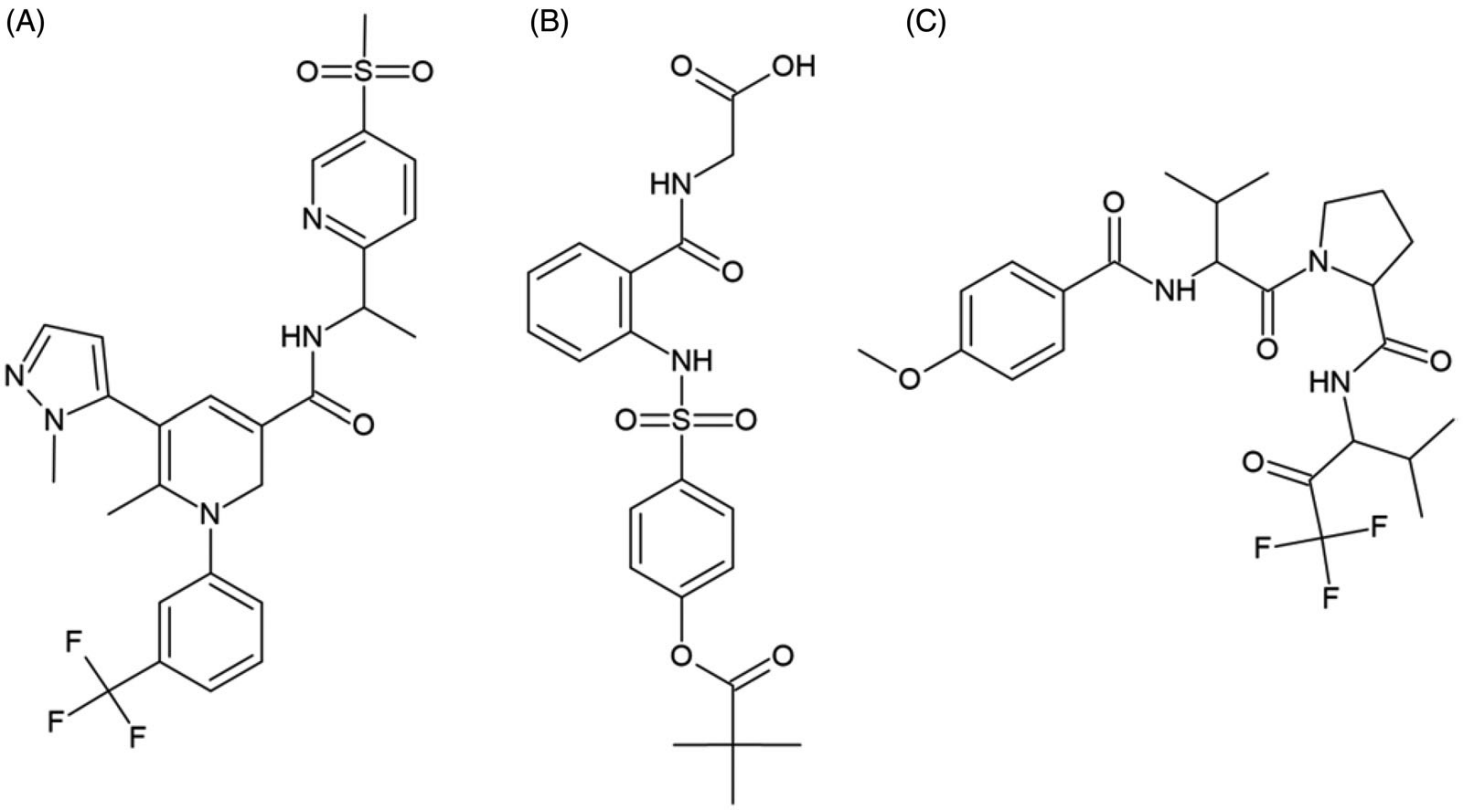
Chemical structures of clinical HNE inhibitors. (A) MPH-966, (B) ONO-5046, and (C) ZD-0892.

**Figure 3.**
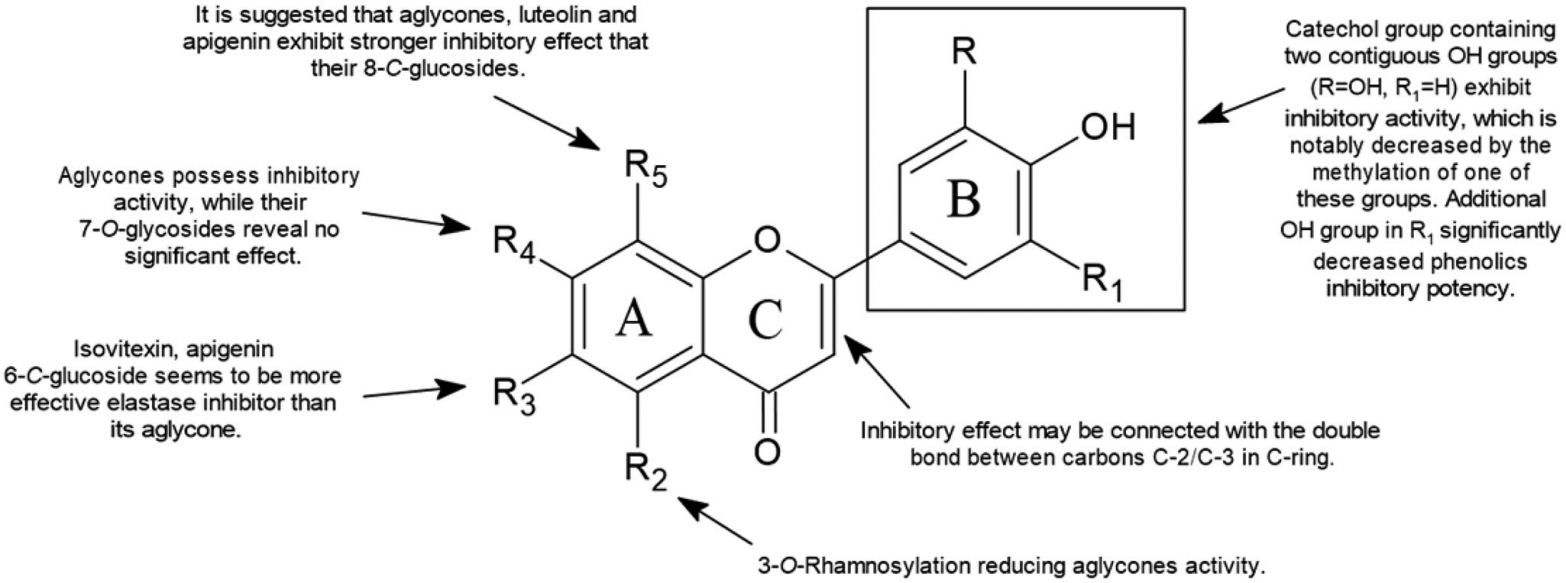
Chemical groups responsible for flavonoid activity (SAR).

Flavonoid SAR analyses enable the determination of the chemical groups responsible for evoking a target biological effect in the organism. The SAR can be used to explain the effect of the structural characteristics of molecules on their activity (Figure 2) and is essential for determining the mechanism underlying drug action^122–124^ (Figure 3).

Special attention was paid to the number, *O*-methylation, *O*-glycosylation of free hydroxyl groups as well as the *C*-glycosylation in position C-6 and C-8 in A-ring. Natural compounds bearing a catechol group containing two contiguous phenolic OH groups (3′,4′-dihydroxy) exhibit inhibitory activity, which is notably decreased by the methylation of one of these groups. Compounds with a lack of catechol groups possess a weak inhibitory effect on elastase action^125^. Among the four investigated flavonoids, quercetin, myricetin, kaempferol, and galangin, the leading inhibitory potency possesses quercetin, followed by myricetin. It is worth mentioning that the additional OH group in the myricetin molecule at the B-ring (C5′) significantly decreased the phenolic inhibitory potency. The kaempferol and galangin without catechol groups did not exhibit significant inhibitory activity. Moreover, it seems that O-methylation in B-ring leads to an increase in this activity. Luteolin 4′-methyl ether (IC_50_ 4.13 µM) possess higher inhibitory potential than luteolin (IC_50_ 6.91–36.01 µM). Moreover, it seems that *O*-methylation in B-ring leads to increase inhibitory activity. Luteolin 4′-methyl ether (IC_50_ 4.13 µM) possess higher inhibitory potential than luteolin (IC_50_ 6.91–36.01 µM)^71^^,^^74^.

The significance of *O*-glycosylation at the A-ring (C7) and C-ring (C3) positions can be observed by comparing the inhibitory levels of apigenin and luteolin and its 7-*O*-glucosides cosmosiin, and cynaroside, respectively. Based on the IC_50_ values, aglycones possess stronger activity while their 7-*O*-glucosides reveal no significant inhibitory effect. It is worth mentioning that 3-*O*-rhamnosylation of quercetin and kaempferol also reduced their activity. The values presented in Table 3 suggest that glucosylation or rhamnosylation at positions C-7 or C-3 presumably produce steric hindrances that prevent molecules from binding to enzymes^126^. In addition, a comparison of an anti-elastase potential of apigenin and apigenin 4′-*O*-*β*-d-glucoside leads to the conclusion that glucosylation of the hydroxyl group in B-ring also reduces its activity^70^^,^^77^.

*C*-glycosylation of the A-ring occurs at the C6 and C8 positions, which are the most typical locations for glycosyl radicals in the flavonoid skeleton. It seems that the aglycones luteolin and apigenin exhibit stronger inhibitory effects than their 8-*C*-glucosides. On the other hand, isovitexin and apigenin 6-*C*-glucoside are more effective elastase inhibitors than their aglycones (see Table 3).

The inhibitory effect may also be connected with the double bond between carbons C-2 and C-3 in the C-ring of flavonoids^125^^,^^127^. It is suggested that double bonds in the C-ring allow for the maintenance of a spatial and practically planar flavonoid skeleton. The saturation of the double bond may result in the presence of an obtuse angle in the flavonoid structure. Previous findings assumed that the almost flat structure of flavonoids is an important factor in enzyme inhibition activity^126^. These conclusions explain the significantly stronger inhibitory activity of apigenin than naringenin (see Table 3).

**Figure 4.**
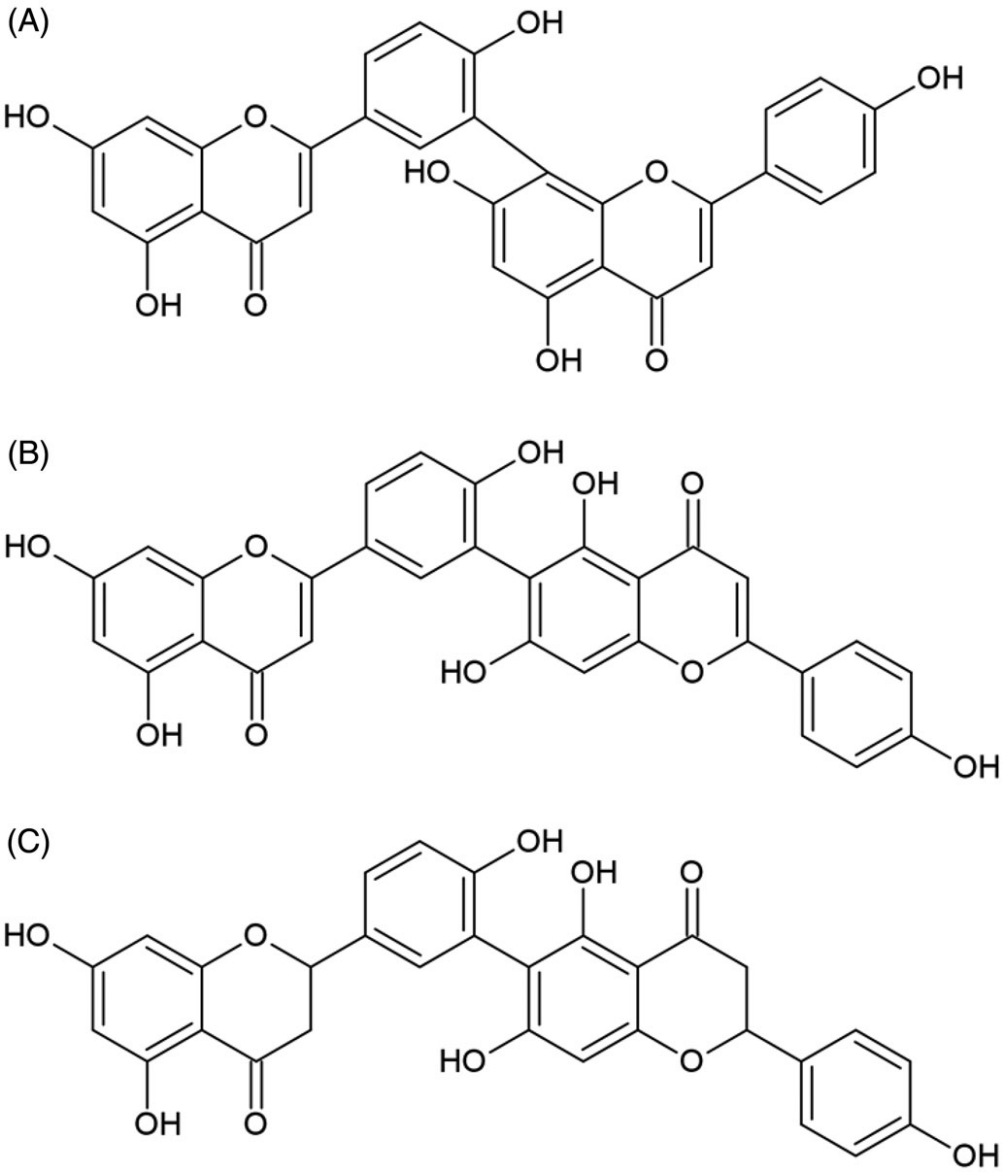
Chemical structures of biflavonoids with anti-elastase potential. (A) Amentoflavone, (B) robustaflavone, and (C) rhusflavonone.

In the group of biflavonoids, anti-elastase activities were examined for cupressuflavone, amentoflavone, robustaflavone, and rhusflavanone (Figure 4)^76^^,^^84^^,^^85^. The amentoflavone and robustaflavone differ in the chromene ring substituent, C-8 and C-6, respectively. The authors observed that their high inhibitory activity might be connected with the optimum number of free hydroxyl moieties. It is worth mentioning that the distinction between chromene ring position does not influence biflavonoids biological effect. The difference in two structures of robustaflavone and rhusflavanone is connected with saturation on double bond in C-ring. Rhusflavanone with a lack of double bond between C-2 and C-3 exhibit much lower inhibitory potential than robustaflavone. Results from this assay were well correlated with those from studies using apigenin and naringenin.

The position of the B-ring in the C-ring allows us to compare flavone and isoflavone activity. Genistein with a 3-B-ring and one hydroxyl group at the A-ring shows similar activity to apigenin, while daidzein with two hydroxyl groups at the A-ring exhibits a stronger effect than flavone. However, the values presented in Table 3 are not sufficient to identify the SAR for this class of compounds.

Summarising, comparison of IC_50_ values allowed pointing out characteristics of flavonoids structures that facilitate their elastase inhibition: catechol structure for B-ring, double bond between C2–C3 at C-ring, *O*-methylation and *C*-glycosylation at A-, B-, and C-ring. The level of plant derivative activity on HNE has been reported to be also connected with the hydrophobicity and molar refractivity of these derivatives, with a bilinear correlation representing the most important relationship^128^. Nevertheless, to correlate the SAR with flavonoid inhibitory effects, additional experiments in different cellular and enzymatic systems must be performed.

## Discussion and conclusions

Significant progress has been made towards discovering natural products as enzyme inhibitors. The high potential of natural compounds lies in their role as lead structures that can be optimised in terms of bioavailability and biological activity. Nevertheless, our knowledge regarding the SAR among the various flavonoid compounds and their impact on elastase action and release is still incomplete. Emerging reports on the activity of various groups of compounds provide information about new elastase inhibitors. On the other hand, the available results yield conflicting information about the level of their inhibitory activity.

To clarify, the authors of this review verified the method criteria for establishing IC_50_ values for compounds presented in Table 3. For example, the difference between IC_50_ values in studies describing luteolin activity may be connected with experiments involving blocking elastase release from neutrophils as well as inhibition of already freed enzymes. It was noted that an IC_50_>300 µM for luteolin activity was established in the test, and it represented the change in absorbance measured after adding enzyme to substrates followed by the incubation process. In comparison, elastase release was measured by degranulation of azurophilic granules and activation of human neutrophils with fMLP. The results are expressed in a fMLP/CB (cytochalasin B)-activated, drug-free control system. In this case, the IC_50_ for luteolin activity reached 6.91 µM. Correspondingly, another flavone commonly found in the plant kingdom, namely, apigenin, has been tested as an elastase inhibitor, and its IC_50_ ranges from 13.35 µM to 46.1 µM. Potent inhibition of HNE release occurs by apigenin after stimulation of cells with fMLP. These results are compatible with data obtained with the use of luteolin as an inhibitor. It was deduced that experiments involving fMLP/CB-stimulated neutrophils showed that apigenin and luteolin were effective. Similar conclusions can also be drawn from the analysis of chrysin IC_50_ values. Superior chrysin activity in human neutrophils was assessed as inhibition of fMLP/CB-induced elastase release (IC_50_=2.44 µM).

Quercetin has been used in many studies as a reference compound with a proven inhibitory effect on elastase. Based on these results, it appears that the value that adequately describes the IC_50_ for quercetin is in the range of 2.6–2.65 µM. However, some researchers established a positive control for this compound at over 300 µM (0.101 mg/mL). In this situation, the distinction between the obtained values seems to be connected with the substrate concentrations (N-succinyl-Ala-Ala-Ala-p-nitroanilide, elastase), pH scale, incubation time, and temperature and the volumes of elastase, inhibitor, and medium solutions.

Moreover, the 5,6,7-trihydroxyflavone baicalein binds not only to the active site but also to the allosteric sites of pancreatic elastase and exhibits a competitive and non-competitive inhibition model, which indicates that the inhibitor molecule may link to either the enzyme–substrate complex or the enzyme alone^80^. According to available data, baicalein exhibits a significant anti-elastase effect (IC_50_=3.53 µM), although research has also indicated a lack of relevant inhibitory activity. The distinction between those extreme results values can be related to different conditions of the conducted experiment. In summary, to specify the ability to inhibit either elastase activity or its release from cells, a wide range of necessary experimental conditions (including substrates, pH level, incubation time, wavelength, volumes, concentrations, and inhibition of enzyme release or free enzyme activity) should be taken into consideration.

The data presented above highlight the diversity of natural phenolic-based structures as elastase inhibitors, thus indicating that novel synthetic inhibitors can be designed and developed based on the structure of phenolic compounds. In practice, a considerable part of every therapy is the selectivity the drug has for its target. On the other hand, compounds may also reveal off-target outcomes due to their toxic and side effects. Anti-target effects follow a narrow level between efficacy and toxicity doses that initiate problems with drug candidate compounds' development. A protein–ligand interaction assessment can be built with *in silico* virtual screening and docking. The available theoretical techniques provide essential information on the compounds and show methods to calculate their binding affinities for the HNE^129^^,^^130^. Structure-shape virtual screening may be practical to identify selective flavonoid inhibitors from databases. Molecular docking is a tool allowing for predicting the potential inhibitory activity and provides a better indication of how a flavonoid can influence its enzyme target^131^. Theoretical methods and computational programmes, including virtual screening, analysis of structure-base and pharmacophore, as well as molecular docking, can be used to pick compounds that target an enzyme and to determine expected targets for well-known and newly discovered phytochemicals^132^. Thus, *in silico* studies can improve successive stages for decreasing off-target effects, activity profiling, and further analysis of natural compounds. It is recommended to establish the efficacy and safety of the described inhibitors using *in vivo* and *in vitro* models, including docking, especially when using such compounds in products to promote health.
